# Dynamics of Attached Bacteria and Potentially Pathogenic Bacteria to Expanded Polystyrene Plastic Litter in Marine Field Experiments

**DOI:** 10.3390/toxics14050392

**Published:** 2026-05-02

**Authors:** Hyun-Jung Kim, Gaeul Jeong, Kang Eun Kim, Jung Hoon Kang, Ok Hwan Yu, Won Joon Shim, Sang Heon Lee, Min-Chul Jang, Jae-Hyeok Lee, Seung Won Jung

**Affiliations:** 1 Library of Marine Samples, Korea Institute of Ocean Science & Technology, Geoje 53201, Republic of Korea; hjkim8845@kiost.ac.kr (H.-J.K.); gaeul@kiost.ac.kr (G.J.); rkddmssl@kiost.ac.kr (K.E.K.); 2 Department of Oceanography and Marine Research Institute, Pusan National University, Busan 46241, Republic of Korea; sanglee@pusan.ac.kr; 3 Department of Ocean Science, University of Science and Technology, Daejeon 34113, Republic of Korea; 4 Ecological Risk Research Department, Korea Institute of Ocean Science & Technology, Geoje 53201, Republic of Korea; jhkang@kiost.ac.kr (J.H.K.); wjshim2000@gmail.com (W.J.S.); 5 Ocean Climate Response & Ecosystem Research Department, Korea Institute of Ocean Science & Technology, Busan 49111, Republic of Korea; ohyu@kiost.ac.kr; 6 Ballast Water Research Center, Korea Institute of Ocean Science & Technology, Geoje 53201, Republic of Korea; mcjang@kiost.ac.kr; 7 Department of Biological Sciences, University of Manitoba, Winnipeg, MB R3T 2N2, Canada; jaehyeok.lee@umanitoba.ca

**Keywords:** bacterial diversity, potentially pathogenic *Vibrio* spp., expanded polystyrene, environmental changes, 16S rDNA metabarcoding

## Abstract

Expanded polystyrene litter in marine environments harbors diverse and distinct microbial communities, referred to as the plastisphere. This study aimed to investigate the monthly dynamics of bacterial and potentially pathogenic bacterial (PPB) communities on expanded polystyrene over one year. *Vibrio* species dominated the PPB community, cooccurring at consistently higher abundances on expanded polystyrene than in the surrounding seawater, particularly under higher temperatures and low dissolved organic carbon (DOC) levels. At a temperature threshold of 16 °C, the abundance of zoonotic species, such as *Vibrio parahaemolyticus* and *Vibrio alginolyticus*, increased significantly. Some psychrotrophic *Vibrio* spp. were detected under moderately eutrophic conditions, suggesting that expanded polystyrene may also serve as a dispersal vector facilitating their transport to more favorable habitats. Multivariate analyses, including partial least squares path modeling, revealed temperature and DOC as the primary environmental factors influencing PPB community composition. However, environmental responses varied by taxonomic groups, with different preferences observed under varying eutrophic conditions. In conclusion, these findings demonstrate that expanded polystyrene litter supports a selective and environmentally responsive bacterial population, highlighting the potential role of plastic debris in promoting pathogenic bacterial persistence and spread in marine ecosystems, particularly under conditions associated with climate change, including warming and eutrophication.

## 1. Introduction

Plastics have become a characteristic feature of the Anthropocene, reflecting the increasing human impact on Earth’s ecosystems and environment. Since the mid-20th century, global plastic production has steadily increased and now exceeds 400 million tons annually [1]. A substantial fraction of this production, estimated at approximately 0.1% to 4% (between 4.8 and 12.7 million tons), enters the oceans [2]. Expanded polystyrene (EPS) accounts for approximately 20% of marine plastic litter and is nearly twice as abundant in regions with intensive aquaculture and coastal activities, particularly in Asia, where it is extensively used in the manufacture of buoys and other buoyant fishing equipment [3]. EPS fragments are more readily converted into microplastics than polypropylene or polyethylene in marine environments [3], owing to their decreased tensile strength. In particular, plastic debris serves as a habitat for complex, persistent microbial communities in marine environments, collectively referred to as the plastisphere [4]. Given its increased fragmentation rate, EPS can more effectively promote the dissemination of pathogenic bacteria than other plastic types, particularly polypropylene [4,5]. Therefore, understanding plastisphere biodiversity is essential for characterizing the microbial taxa colonizing plastic surfaces, their spatial and temporal distribution, and their ecological functions [3].

Plastisphere communities perform distinct metabolic and biogeochemical functions that differ markedly from those of planktonic microbes in the surrounding seawater [6,7]. Colonization begins with bacterial biofilm formation, which initiates ecological succession, shapes community structure, and facilitates the recruitment of diverse marine taxa [8]. In particular, microbial abundance and diversity shift rapidly within the first one to two weeks, reflecting a transition from primary to secondary colonizers. Early-stage colonization is strongly influenced by environmental conditions and surface roughness [9]. In later stages, bacterial community assembly becomes less dependent on deterministic factors, such as specific taxa recruitment or quorum-sensing-mediated expansion, and more influenced by stochastic colonization events, nutrient dynamics, environmental pressures, and microbial interactions [10]. Over time, these successional dynamics develop into a mature, self-organizing microbial habitat that is ecologically distinct from the surrounding seawater. Moreover, the plastisphere harbors a diverse array of pathogenic bacteria, including *Vibrio*, *Acinetobacter*, and *Clostridium*, which are typically more abundant on plastic surfaces than in the surrounding seawater [11,12]. These pathogens can be transmitted through marine trophic networks, from zooplankton to marine animals, posing potential risks to human health [13,14,15]. The persistence of potentially pathogenic taxa within these communities underscores both the ecological significance of the plastisphere and its potential public health implications.

Despite increasing research on plastisphere communities, the early colonization and succession of pathogenic bacteria, including early attachment and succession processes, on EPS in natural marine environments, remain poorly understood. This knowledge gap impedes accurate prediction of plastisphere dynamics and assessment of potential risks associated with plastic pollution. Moreover, knowledge of the diversity, temporal dynamics, and environmental drivers of pathogenic bacteria within the plastisphere remains limited. Therefore, the mechanisms underlying biofilm establishment and microbial succession on plastics require further investigation. In the present study, we investigated the early temporal dynamics of bacterial and potentially pathogenic communities colonizing EPS in a natural marine environment. To this end, we examined the influence of environmental parameters on the structure and composition of EPS-associated bacterial communities by conducting monthly monitoring during the initial stages of colonization. The primary objectives were to (i) identify early colonizers and plastic-specific taxa, (ii) evaluate the influence of environmental factors on plastisphere assembly, and (iii) assess the abundance of *Vibrio* species and their response to environmental variables, such as temperature, dissolved organic carbon (DOC), and eutrophication index (EI). This study addresses key knowledge gaps concerning the early development of the plastisphere and its potential function as a reservoir for pathogenic bacteria within marine ecosystems.

## 2. Materials and Methods

### 2.1. Study Area and Sample Collection

**Figure 1:**
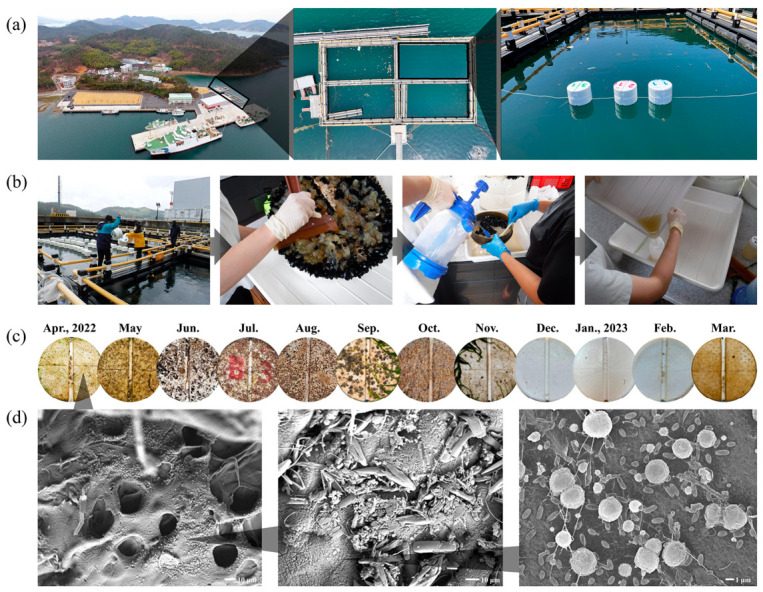
Overview of the experimental procedures and microbial colonization on expanded polystyrene. (**a**) In situ experimental setup for observing initial bacterial attachment on expanded polystyrene. (**b**) Procedure for detaching surface-associated bacterial communities from expanded polystyrene. (**c**) Photographic representation of the monthly temporal dynamics of microbial colonization on expanded polystyrene surfaces. (**d**) Scanning electron microscopy images depicting bacterial attachment on expanded polystyrene surfaces.

In this study, EPS substrates were selected, as they are widely used in shellfish aquaculture and represent prevalent plastic debris subject to microbial colonization in the study area [3]. Therefore, an in situ field experiment was conducted using a commercial EPS buoy at the Jangmok Bay Time-series Monitoring Site (JBTMS; 34°59′37″ N, 128°40′27″ E), Republic of Korea [9,16,17], from 29 May 2022 to 28 May 2023 (Figure 1a). Three replicate EPS substrates, cylindrical with a radius of 17 cm, were exposed at the sea surface for one month and subsequently retrieved. Concurrently, 2 L of surrounding seawater was sampled for environmental and metabarcoding analyses. The EPS substrates were secured to ensure that only one side was exposed to seawater (Figure 1a). During sampling, retrieved buoys were immediately transferred to an onboard laboratory located within 10 m of the site to prevent desiccation. In the laboratory, each EPS substrate was gently rinsed with sterilized seawater to remove dust, and eukaryotic plankton and large attached organisms, such as macroalgae, were carefully removed using sterilized forceps. The initial biofilm was collected by brushing a 10 cm × 10 cm area of the EPS surface in contact with seawater with a soft brush under sterilized seawater. The collected biofilm, including bacteria and eukaryotes, was transferred into a sterilized 200 mL polyethylene bottle, and the volume was adjusted to 100 mL with sterilized seawater (Figure 1b). Samples were centrifuged at 1800× *g* for 10 min to concentrate organisms for genomic DNA (gDNA) extraction. For comparison, 1 L of seawater was filtered through a 0.2 µm polycarbonate filter (GTTP 04700, QIAGEN, Hilden, Germany) to concentrate bacterial cells. Pellets and filters were stored at −80 °C for subsequent metabarcoding. Bacterial colonization on EPS surfaces was assessed using scanning electron microscopy (SEM; model JSM-5600LV; Jeol, Tokyo, Japan), as previously described [18].

### 2.2. Analysis of Bacterial Community

Bacterial metabarcoding was conducted as described in our previous studies [9,12,16]. Briefly, approximately 400 mg of EPS-derived pellets and filters containing concentrated seawater bacteria were placed in 1.5 mL microcentrifuge tubes for gDNA extraction using the DNeasy PowerSoil Pro Kit (QIAGEN, Hilden, Germany). Extracted DNA was standardized to 20 ng, and the V3–V4 regions of the 16S rDNA gene were amplified using universal primers 341F and 800R tagged with Illumina adapters (Illumina Inc., San Diego, CA, USA) (Supplementary Table S1). Polymerase chain reaction (PCR) products were purified (QIAquick PCR Purification Kit; QIAGEN) and indexed in a second PCR using the Nextera XT 96 index kit v2 (Illumina Inc., San Diego, CA, USA). DNA quantification was performed with a Bioanalyzer 2100 (Agilent Technologies, Santa Clara, CA, USA). Amplicons were normalized, pooled, and sequenced on the Illumina MiSeq platform (Illumina Inc.).

After sequencing, denoised sequences were analyzed in Quantitative Insights Into Microbial Ecology 2 (QIIME2, version 2022.04) [19], excluding non-bacterial sequences, including archaea, mitochondrial, and chloroplast DNA. Amplicon Sequence Variants (ASVs) were generated using the Deficiency of Adenosine Deaminase 2 pipeline (DADA2, https://github.com/benjjneb/dada2, accessed on 10 January 2025), which enables high-resolution inference of sequence variants while minimizing the risk of false positives [20]. The DADA2 algorithm was applied for quality filtering, trimming, denoising, paired-end merging, and chimera removal (Supplementary Table S2). Although 16S rDNA metabarcoding is a powerful approach for characterizing bacterial community composition, its resolution for species-level classification remains limited, particularly among closely related taxa [12]. Accordingly, in this study, species-level assignments of PPB were reported alongside the corresponding ASV information to improve taxonomic resolution and traceability. Representative pathogenic ASVs and their sequence identities are provided in Supplementary Table S3.

### 2.3. Identification of Potentially Pathogenic Bacterial (PPB) Community

A comprehensive list of PPB was compiled by integrating information from multiple authoritative sources. The initial dataset was obtained from the Eukaryotic Pathogen, Vector, and Host Informatics Resources (VEuPathDB, https://veupathdb.org), which includes records on over 600 taxa encompassing eukaryotic pathogens, including protists and fungi, invertebrate vectors, and various pathogenic and non-pathogenic organisms [21]. The Enhanced Infectious Disease Database (EID2, https://eid2.liverpool.ac.uk) was also used, owing to its extensive repository of host–pathogen associations and interaction data. Relevant literature, including Bacterial Pathogenesis and Bacterial Pathogens of Marine Fish [22,23], was reviewed to supplement the list. The criteria used to extract PPB from the metabarcoding dataset generated in this study are detailed in Supplementary Table S3, with cross-referencing to the databases.

### 2.4. Measurement of Environmental Factors

Environmental parameters were measured according to our previously established protocols [9,12,16]. After seawater collection, temperature, salinity, pH, and dissolved oxygen (DO) were measured using a YSI EXO2 Sonde probe (Xylem Inc., Washington, DC, USA). For dissolved inorganic nutrients, including dissolved inorganic nitrogen (DIN; NO_2_^−^, NO_3_^−^, and NH_4_^+^), dissolved inorganic phosphorus (DIP), and dissolved silica (DSi), 50 mL of seawater was filtered through a 0.2 µm polycarbonate filter. The filtrate was analyzed using an automated nutrient analyzer (QuAAtro39; SEAL Analytical, Mequon, WI, USA). To determine chlorophyll-a (Chl-*a*) concentrations, 1 L of surface seawater was filtered through a 47-mm glass fiber filter (GF/F; Whatman plc, Maidstone, UK) under low vacuum pressure. The filter was soaked in 10 mL of a 90% acetone-distilled water solution and stored in the dark at 4 °C for 24 h to extract phytoplankton pigments. Chl-*a* was quantified using a 10-AU fluorometer (Turner Designs, Inc., Sunnyvale, CA, USA). DOC was measured by filtering 50 mL of each sample through a pre-combusted (450 °C for 12 h) GF/F filter using gravity filtration. DOC concentrations were determined using high-temperature catalytic oxidation (Shimadzu, Kyoto, Japan). All measurements were performed in triplicate to ensure analytical reliability. The resulting triplicate values (or duplicates, where applicable) were averaged for statistical analysis.

The eutrophication index (EI) at each sampling date was calculated based on the concentrations of dissolved inorganic nutrients and Chl-*a* using the following equation [24,25]:

(1) $$\mathrm{EI}=0.279C_{{\mathrm{PO}}_{4}}+0.261C_{{\mathrm{NO}}_{3}}+0.296C_{{\mathrm{NO}}_{2}}+0.275C_{{\mathrm{NH}}_{3}}+0.214C_{\text{Chl-}a}$$

Modified classification thresholds for Korean coastal waters were used as previously described [12], with values categorized as low (<1), moderate (1–3), and high (>3).

### 2.5. Statistical Interpretation of the Obtained Data

Prior to statistical analysis, environmental variables and bacterial ASVs were square root-transformed to minimize data skewness and enhance the signal-to-noise ratio [7], and the transformed environmental data were then normalized. Bacterial community structures, including PPB, were assessed using the Bray–Curtis dissimilarity followed by hierarchical clustering. Differences in communities associated with environmental parameters (e.g., water temperature and EI) were evaluated using non-metric multidimensional scaling (NMDS) based on group averages, and statistical significance was tested using permutational analysis of variance (PERMANOVA) with 999 permutations [26]. Pearson correlation analysis was performed to assess the relationship between bacterial taxonomic composition and diversity. All analyses were performed using PRIMER 7+ (version 7+; Primer-E Ltd., Plymouth, UK). 

Alpha diversity metrics, including the Shannon index, were computed using the “vegan” package (version 2.7-1) in R software (version 4.3.2, R Foundation for Statistical Computing, Vienna, Austria) [27]. Heatmaps visualizing dominant ASV distribution patterns were generated using ggplot2 (version 3.5.1) [28]. All statistical analyses and graphical representations were conducted in the R Studio environment. Linear discriminant analysis effect size (LEfSe), which is suitable for high-dimensional datasets, was applied to detect taxa that significantly distinguish bacterial communities across groups [29]. The LEfSe workflow began with a non-parametric Kruskal–Wallis test to detect significantly varying taxa across classes, followed by Wilcoxon rank-sum tests to assess subclass consistency using an alpha threshold of 0.05 [30]. Taxa that were both statistically significant and consistently enriched were further evaluated using linear discriminant analysis (LDA) to estimate the effect sizes. LEfSe was implemented via the microbiomeMarker package (version 1.13.2) [31], enabling biomarker discovery in microbial datasets. To visualize the taxonomic distribution and relative abundance of differentially abundant taxa, a heat tree was generated using the metacoder package (version 0.3.8) [32]. Taxonomic data derived from the LEfSe were formatted into a hierarchical structure and loaded into a tax map object. The heat_tree function was used to map statistical significance and LDA scores onto a phylogenetic tree, with node size representing taxon abundance and color intensity indicating effect size, facilitating intuitive comparisons.

Random Forest modeling was employed, as it effectively accommodates complex and nonlinear ecological data, making it suitable for predictive analyses of microbial communities. Predictive modeling was conducted using the random forest package in R (version 4.7.1.2) [33], with the number of trees (ntree) set to 500 and the default node size (nodesize) of 5. The optimal number of variables per split (mtry) was determined through repeated 10-fold cross-validation using the caret package. The model was optimized to minimize the root-mean-square error (RMSE) and maximize the coefficient of determination (R^2^), ensuring robust predictions. The final model was trained on the complete dataset using the optimal mtry [34]. To assess the influence of environmental variables on the abundances of PPBs, variable importance scores were calculated to determine the relative influence of each predictor on the performance of the model in predicting PPB abundance. Model performance was evaluated using RMSE, R^2^, and the Nash–Sutcliffe efficiency coefficient (NSEC); models with RMSE near zero and R^2^ and NSEC values approaching one were considered highly predictive.

Associations between prevalent PPB taxa and environmental variables were investigated using the MaAsLin2 package in R (version 1.20.0) [35]. As this tool implements a generalized linear model framework with stepwise normalization, it addresses challenges specific to microbiome data, including sparsity and compositionality. PPB-associated ASVs were normalized using total sum scaling (TSS) and log-transformed to stabilize the variance. Environmental variables (e.g., temperature, salinity, DO, pH, DOC, and EI) were included as fixed effects, whereas sampling day was treated as a random effect. Significant associations were identified through linear modeling. *p*-values were adjusted using the Benjamini–Hochberg false discovery rate (FDR) method, with significance set at *p* < 0.05. Complementary non-parametric tests, such as the Wilcoxon rank-sum test and Spearman correlation, were also applied. Features with FDR-adjusted q ≤ 0.25 were considered significant [35]. Model outputs included effect estimates, standard errors, and q-values, enabling the identification of PPB taxa.

Partial least squares path modeling (PLS-PM, version 0.5.1) [36] was applied to examine direct and indirect effects of environmental variables on the composition and interactions of PPB and *Vibrio* spp. PLS-PM was selected for its ability to handle complex relationships among latent variables and accommodate multicollinearity commonly present in ecological datasets [37]. Analyses were performed using the PLS-PM package in R, with permutation tests (100 iterations) conducted to assess model robustness. Key environmental predictors were identified using multiple linear regression and Spearman correlation. Variables with loading values ≥ 0.7 were retained in the structural equation model to ensure interpretability [38]. Path coefficients quantified relationships between microbial and environmental variables, and model fit was evaluated using the goodness-of-fit (GOF) index.

## 3. Results

### 3.1. Dynamics of Bacterial Community and PPB Community

**Figure 2:**
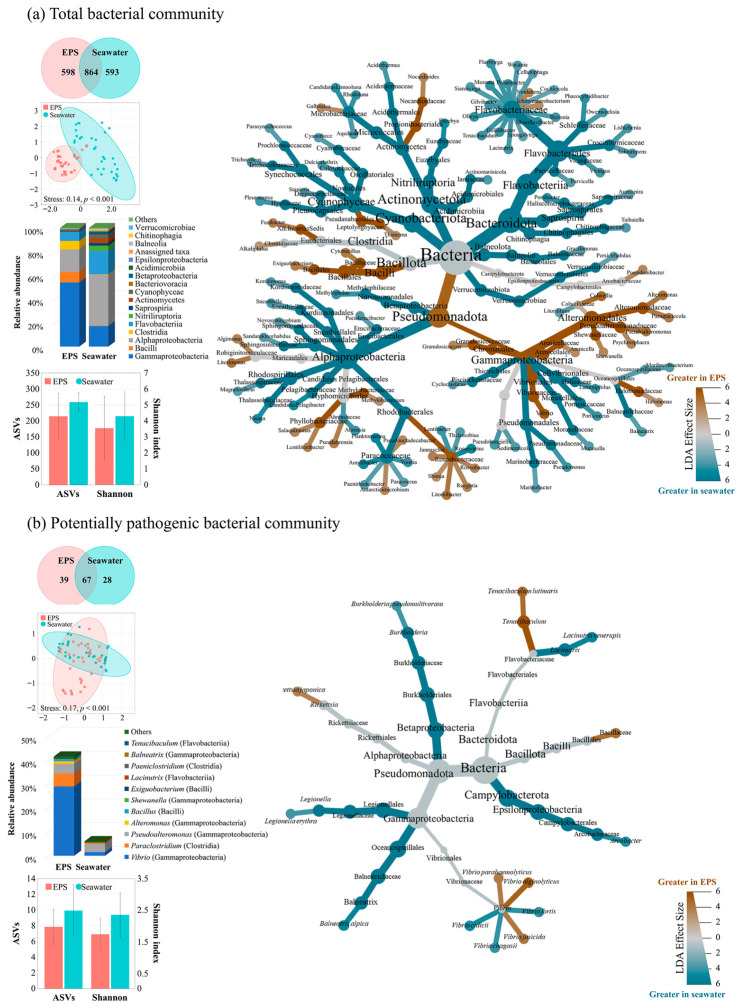
Dynamics of the total bacterial community and the potentially pathogenic bacterial (PPB) community. (**a**) Species-level Venn diagrams, non-metric multidimensional scaling (NMDS), relative abundance, amplicon sequence variants (ASVs), Shannon diversity indices, and a heat tree diagram based on linear discriminant analysis effect size (LEfSe) are presented for the total bacterial community. The heat tree illustrates taxonomic differences aggregated at the genus level, with color indicating relative enrichment in either expanded polystyrene (brown) or seawater. Grey branches indicate taxa with no statistically significant differences between substrates. (**b**) Comparative analysis of PPB on expanded polystyrene and in seawater, including a heat tree diagram visualizing taxonomic differences at the species level.

Representative images depicting biofilm formation on the EPS surface each month are presented in Figure 1c. The increase in water temperature from May to September coincided with an increase in the fouling bacterial communities rather than diverse biofilm communities. SEM analysis of the EPS surface revealed initial bacterial colonization, characterized by rod-shaped and coccoid bacteria-like cells (Figure 1d). Bacterial metabarcoding of EPS and surrounding seawater identified a total of 2055 ASVs, including 1462 and 1457 ASVs associated with EPS and seawater, respectively (Figure 2a; Supplementary Table S2).

Community composition differed significantly between EPS and seawater samples (PERMANOVA, pseudo-F = 4.71, *p* < 0.001). Specifically, throughout most of the study period, bacterial communities on the EPS surface were dominated by Gammaproteobacteria (54.1%), whereas Alphaproteobacteria (18.9%) and Flavobacteriia (7.6%) became dominant during colder months (January–February). The abundance of Bacilli (8.9%) and Clostridia (7.2%) increased with rising temperatures (August–September) (Supplementary Figure S1). In contrast, Alphaproteobacteria consistently accounted for over 40% of the seawater bacterial community across most sampling periods. Moreover, Shannon diversity was lower on EPS than in seawater; however, it increased in parallel with the relative abundances of Alphaproteobacteria and Flavobacteriia. Particularly, EPS diversity exhibited a significant negative correlation with Gammaproteobacteria abundance (r = −0.71, *p* < 0.01).

LEfSe analysis identified 43 genera significantly enriched on EPS and 63 genera associated with seawater, indicating distinct bacterial communities between the two substrates (Figure 2a, Supplementary Table S4). Specifically, EPS-enriched taxa were predominantly members of Pseudomonadota, particularly the orders Alteromonadales and Vibrionales within Gammaproteobacteria, along with Bacilli. Specific lineages of Campylobacterota and Clostridia were also characteristic of EPS communities, whereas Alphaproteobacteria and Bacteroidota were dominant in seawater. Moreover, a total of 106 and 95 PPB taxa were identified on EPS and in seawater, respectively. Among these, 39 PPB taxa were unique to EPS, whereas none were exclusive to seawater (Figure 2b). PPB communities significantly differed between the substrates (PERMANOVA, pseudo-F = 2.90, *p* < 0.001), representing an average of 41.6% of the EPS bacterial community compared to only 6.0% in seawater. *Vibrio* spp. represented the most abundant PPB genus, peaking from May to July, with a mean relative abundance of 29.2%. *Pseudoalteromonas* (4.1%) peaked in August, while *Paraclostridium* (5.4%) was more abundant in September (Supplementary Figure S2). The Shannon diversity pattern of the PPB community mirrored that of the total bacterial community. In particular, LEfSe analysis identified five PPB taxa as EPS-specific biomarkers, including *Vibrio alginolyticus*, *V. jasicida*, *V. parahaemolyticus*, *Tenacibaculum lutimaris*, and *Rickettsia japonica*. Conversely, *V. cidicii*, *V. fortis*, and *V. chargasii* emerged as seawater-specific biomarkers (Figure 2b, Supplementary Table S4). Among the 57 *Vibrio* ASVs detected, 38 were characterized as potentially pathogenic (Supplementary Figure S2). EPS was primarily dominated by *V. alginolyticus*, *V. chemaguriensis*, *V. cyclitrophicus*, *V. gigantis*, *V. litoralis*, *V. pelagius*, and *V. parahaemolyticus*, whereas *V. kanaloae* predominated in seawater. Although some *Vibrio* species were shared between the two environments, their temporal distribution and relative abundances differed significantly. For example, several dominant *Vibrio* species, including *V. alginolyticus, V. chemaguriensis, V. pelagius*, and *V. parahaemolyticus*, exhibited significantly different distribution patterns between EPS and seawater (pseudo-F = 2.68, *p* < 0.05).

### 3.2. Environmental Drivers of the PPB on EPS

**Figure 3:**
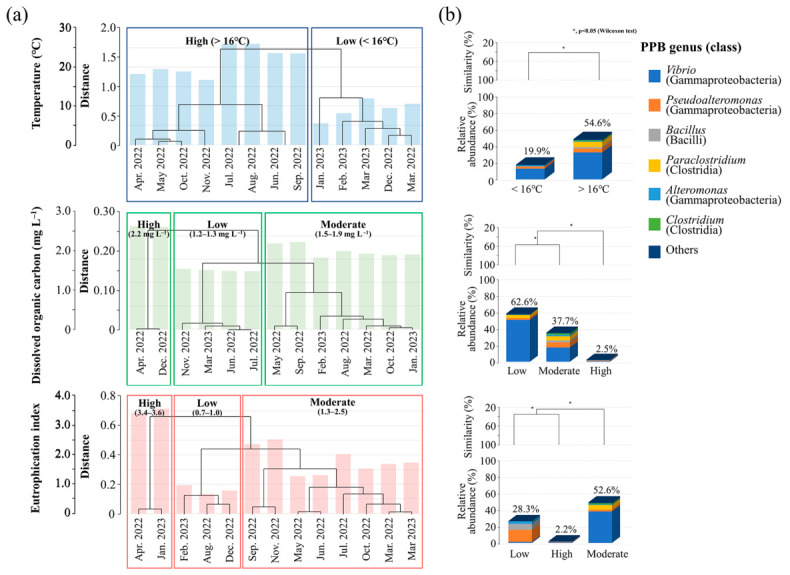
Influence of key environmental parameters on the distribution of potentially pathogenic bacterial (PPB) communities on expanded polystyrene. (**a**) Sampling periods grouped by environmental categories based on temperature, dissolved organic carbon (DOC), and eutrophication index. (**b**) Average relative abundance of PPB taxa across the environmental categories defined in (**a**) and hierarchical clustering of community composition.

During the study period, environmental conditions varied considerably (Figure 3a; Supplementary Figure S3). Specifically, the water temperature ranged from 5.5 to 26.0 °C, while DOC concentrations varied between 1.2 and 2.2 mg L^−1^. The EI, which was calculated based on DIN, DIP, and Chl-*a*, ranged from 0.7 to 3.6. Moreover, cluster analysis using Euclidean distance revealed distinct environmental groupings, including a temperature threshold at 16 °C and three clusters for DOC and EI (Figure 3a).

**Figure 4:**
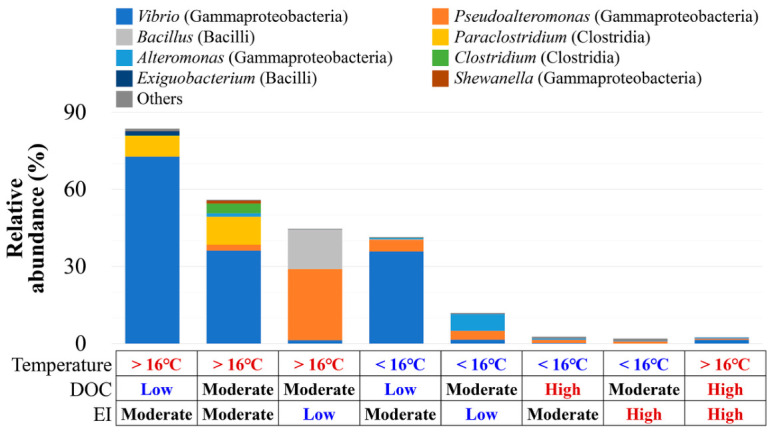
Shifts in the composition of potentially pathogenic bacterial (PPB) community taxa across combined environmental categories. Environmental groupings are defined by temperature, dissolved organic carbon (DOC), and eutrophication index (EI), as identified in Figure 3.

At temperatures exceeding the 16 °C threshold, PPB constituted 54.6% of the total community, representing a 2.7-fold increase compared to that observed under lower temperatures (Figure 3b). Most dominant genera thrived under warmer conditions, except for *Alteromonas*. Under low DOC, PPB abundance was the highest at 62.6%, whereas it decreased to 2.5% under high DOC. PPB peaked at moderate EI (52.6%), followed by low (28.3%) and high EI (2.2%). *Vibrio* dominated the community at moderate EI alongside *Paraclostridium* and *Clostridium*, whereas *Pseudoalteromonas*, *Alteromonas*, and *Bacillus* were more abundant at low EI. Importantly, *Vibrio* accounted for over 60% of PPB under warm temperatures, low DOC, and moderate EI (Figure 4; Supplementary Figure S4), with *Exiguobacterium* exhibiting similar preferences. *Pseudoalteromonas* and *Bacillus* peaked at warm temperatures with moderate DOC and EI, whereas *Paraclostridium* and *Clostridium* preferred warm temperatures with moderate DOC and EI. *Alteromonas* were more frequent at lower temperatures. In particular, at moderate EI, *V. alginolyticus* was detected even at temperatures below 16 °C (Supplementary Figure S4). Overall, these patterns suggest that dominant PPB respond differently to environmental conditions, with their abundance markedly reduced under high DOC or EI.

**Figure 5:**
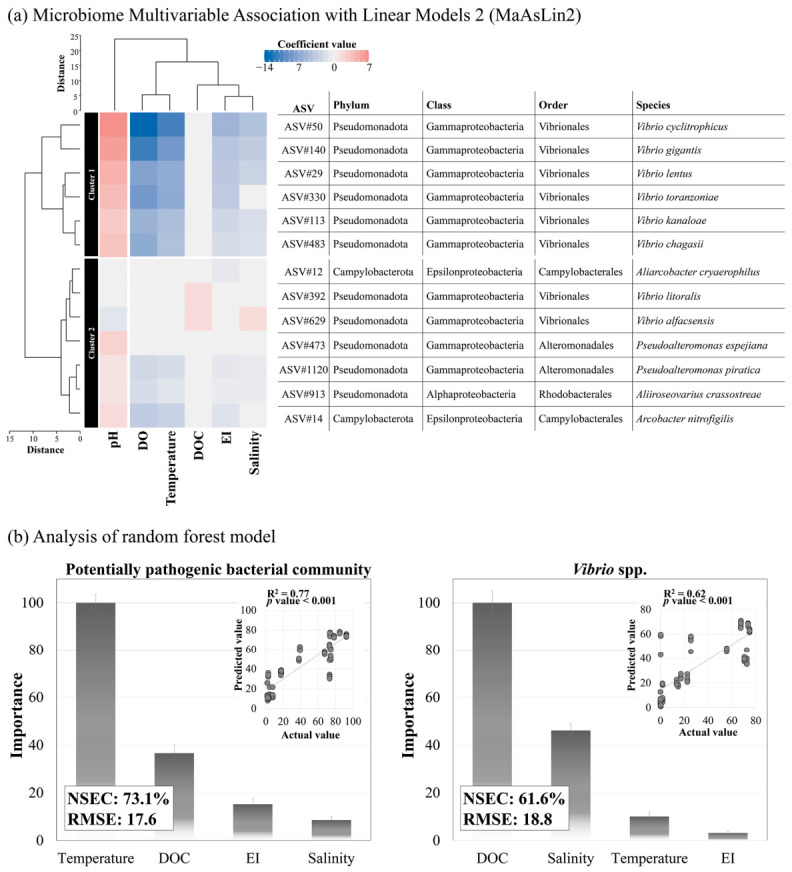
Associations between environmental variables and the potentially pathogenic bacterial (PPB) community. (**a**) Heatmap, generated using multivariable association with linear models 2 (MaAsLin2), illustrates significant associations between PPB taxa and environmental variables, including pH, dissolved oxygen (DO), temperature, dissolved organic carbon (DOC), eutrophication index (EI), and salinity. PPB taxa are clustered into two groups based on their environmental response profiles. (**b**) Random forest analysis depicts the relative importance of environmental factors in shaping the PPB and *Vibrio* spp. The upper-right panels display regression plots comparing predicted and observed values, with model performance evaluated using the coefficient of determination (R^2^), Nash–Sutcliffe efficiency coefficient (NSEC), and root-mean-square error (RMSE). The lower panel depicts the ranked importance of each environmental variable.

MaAsLin2 analysis revealed 13 PPB-associated ASVs grouped into two clusters, each characterized by distinct environmental responses (Figure 5a; Supplementary Table S5). Cluster One (Cluster 1) consisted solely of *Vibrio* ASVs that were strongly correlated with multiple environmental variables except DOC. Cluster 2 included various bacteria, with some *Vibrio* species exhibiting positive DOC associations. Moreover, random forest modeling significantly identified DOC and temperature as primary drivers of *Vibrio* and PPB composition, respectively (*p* < 0.001), with EI exhibiting only minor influence (Figure 5b). The *Vibrio* and PPB models demonstrated high prediction accuracy, with NSECs evaluated at 73.1% (RMSE 17.6%) and 61.6% (RMSE 18.8%), respectively.

**Figure 6:**
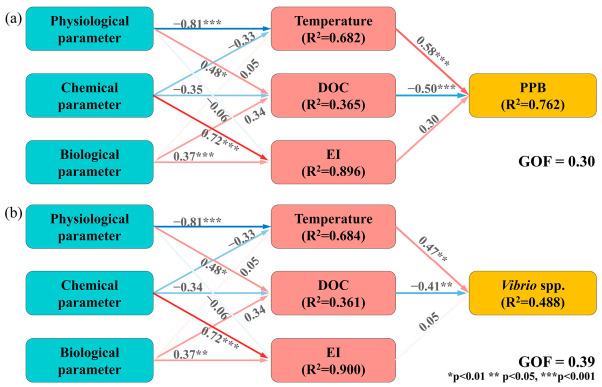
Partial least squares path modeling (PLS-PM) illustrates the influence of environmental factors on (**a**) the potentially pathogenic bacterial community (PPB) and (**b**) *Vibrio* spp. Arrows indicate the direction of relationships between exogenous and endogenous variables. Red lines denote positive associations, while blue lines indicate negative ones. The strength of each association is reflected by color saturation: deeper red indicates stronger associations, while lighter shades indicate weaker ones. R^2^ values represent the proportion of variance explained for each endogenous variable. Model fit was evaluated using the goodness-of-fit (GOF) index. Asterisks indicate significant path coefficients.

We assessed environmental impacts on PPB and *Vibrio*, including physical (temperature, salinity, DO, and pH), chemical (DIN, DIP, and DOC), biological (Chl-*a*) factors, and the EI variable (Figure 6), using PLS-PMs. Our results indicated that the PPB model demonstrated a good fit and explanatory power (GOF = 0.30, R^2^ = 0.762). The *Vibrio* model exhibited a better overall fit (GOF = 0.39) but accounted for a lower proportion of variance explained (R^2^ = 0.488), suggesting influence from unmeasured factors. Moreover, EI exhibited the highest R^2^, followed by temperature and DOC. Both models confirmed temperature and DOC as significant predictors.

## 4. Discussion

In the marine environment, plastic debris provides a unique substrate for the formation of long-lasting and complex microbial communities [4]. This plastisphere may serve as a reservoir for pathogens, raising public health concerns [12]. Building on a previous study on bacterial community diversity in the marine plastisphere [20], in the present study, we examined the dynamic responses of microbial assemblages in a large-scale field experiment, particularly PPBs, to environmental fluctuations on EPS surfaces. In particular, this approach overcomes the limitations of field-collected samples, which often lack clear information on residence time, exposure, and transport routes. Our results confirm significant differences in bacterial communities and PPBs between the surrounding seawater, consistent with our previous studies [9,12]. The presence of EPS-specific PPBs, particularly *Vibrio* spp., suggests that the plastisphere serves as a selective substrate or shelter for microorganisms from the seawater. This selective colonization is strongly influenced by environmental conditions, indicating that microbial enrichment on plastics is driven by environmental filtering rather than stochastic processes [39,40], leading to marked differences in microbial composition between the EPS and surrounding seawater. Our results also indicated that Gammaproteobacteria dominated the PPB community on EPS, whereas Alphaproteobacteria was dominant in seawater, consistent with the results of Frère et al. [41]. As primary colonizers, Gammaproteobacteria attach to plastic surfaces via motility and extracellular polymeric substance production [7]. Although they originate from seawater, these organisms establish selective microhabitats on plastics [9], resulting in distinct communities compared to the surrounding seawater [42].

The genus *Vibrio* is a dominant pathogenic member of early-colonizing plastisphere communities [4,9], and *Vibrio* species exhibit a feast-or-famine lifestyle that supports rapid colonization and efficient resource utilization on plastic surfaces [9,39]. Their presence in ingested plastics may increase infection risks for marine oyster aquaculture [43]. Moreover, the consistent detection of *Vibrio* spp. as early plastic colonizers highlights their potential pathogenicity [4,12,17]. In this study, we identified 39 PPB species, including 11 *Vibrio* species, exclusively in EPS biofilms. Motility and chemotaxis, which facilitate host colonization, also contribute to biofilm development under favorable conditions [44,45]. Moreover, the expression of biofilm- and virulence-associated genes in species such as *V. coralliilyticus* and *V. diabolicus* is influenced by environmental factors, particularly seawater temperature [44,46]. Therefore, these findings emphasize that environmental factors influence bacterial survival and community assembly on plastic surfaces, contributing to the development of unique, niche-adapted microbial taxa [47]. In addition, the dominance of *Vibrio* on EPS surfaces highlights the substantial role of environmental factors in shaping microbial colonization within the plastisphere. These findings are strongly supported by short-term in situ experiments [9] and a large-scale field analysis of approximately 600 plastic debris samples [12], providing robust evidence for environment-driven bacterial selection. For example, Kim et al. reported that water temperature, DOC, and EI are key environmental factors influencing the PPB community within the plastisphere [12]. In our study, we identified a water temperature of 16 °C as a critical threshold at which *Vibrio* spp. exhibited increased abundance. Consistently, previous studies indicated that enhanced abundance of zoonotic pathogens, such as *V. parahaemolyticus*, *V. alginolyticus*, *V. chemaguriensis*, and *V. pelagius*, at a water temperature exceeding 16 °C [48,49,50,51]. These observations support concerns that rising sea surface temperatures associated with climate change may favor the proliferation of pathogenic *Vibrio* spp. in marine ecosystems [52,53,54]. However, some *Vibrio* species are psychrotrophic and can grow under cold conditions [55]. In our study, cluster analysis revealed that members of Cluster 1 are adapted to low temperatures, consistent with the findings of Vezzulli et al. [56]. In cold environments, psychrotrophic *Vibrio* may enhance biofilm formation as a survival strategy, potentially mediated by temperature-induced changes in cell surface hydrophobicity [57,58]. Consequently, EPS may promote the dispersal of cold-adapted species, such as *V. alginolyticus*, allowing them to persist in low-temperature waters while proliferating under favorable conditions [58]. Consistent with the ecological concept that “everything is everywhere, but the environment selects” [59], these results suggest that environmental factors, rather than geographic barriers, govern *Vibrio* biogeography. The strong responsiveness of *Vibrio* to environmental variability highlights the need for a deeper understanding of its ecological dynamics amid changing oceanic conditions. Moreover, the expansion of *Vibrio* habitats is closely linked to the combined impacts of the global rise in plastic pollution and climate change [60]. Therefore, continuous monitoring of *Vibrio* in the plastisphere is essential for assessing long-term ecological consequences and emerging public health risks.

In the present study, DOC and EI emerged as key environmental drivers, with moderate EI and low DOC levels favoring PPB abundance. These results are consistent with previous studies highlighting the strong association between PPB and the availability of dissolved organic matter (DOM), such as DOC, Chl-*a*, and dissolved nutrients [13]. Specifically, Jeong et al. [9] and Kesy et al. [61] reported that the adaptation of PPB is largely attributed to their rapid surface colonization, enabling these bacteria to exploit DOM from diverse marine sources, including bivalves, macroalgae, and microalgae, even under nutrient-limited conditions. Kim et al. [12] also reported a strong association between EI and PPB association; however, direct comparison with our results is not feasible, as their study area exhibited higher EI levels than our study site. In our study, moderate EI levels coincided with the summer season, suggesting that *Vibrio* proliferation may be influenced by both temperature and seasonal increases in EI. Unlike in seawater, DOC in the plastisphere may promote antagonistic interactions by enhancing intra- and interspecific bacterial competition [62]. Variability in *Vibrio* responses to DOM may reflect substrate- or habitat-specific strategies, as environmental factors and their interactions vary across contexts [63,64]. Although PPB abundance is influenced by environmental factors, our model accounted for only part of the observed variability, possibly owing to the physicochemical properties of plastics that influence bacterial attachment and subsequent community development [65]. On plastic surfaces, early colonizers likely shape community structure through microbial interactions [7], suggesting that plastisphere dynamics may substantially contribute to community assembly in addition to environmental filtering processes.

## 5. Conclusions

This study demonstrates that EPS in marine environments selectively enriches pathogenic *Vibrio* species. Both field observations and in situ experiments support the role of environmental filtering in influencing plastisphere microbial communities. *Vibrio* species, which are opportunistic pathogens, exhibit a feast-or-famine lifestyle that enables rapid colonization and efficient DOM utilization on plastic surfaces. Their abundance of EPS increased from June to October, corresponding with rising seawater temperatures and indicating clear niche specialization. These findings support the concept of environmental selection, where abiotic conditions primarily determine microbial community composition. Moreover, the expansion of *Vibrio* habitats aligns with escalating plastic pollution and climate change, highlighting the importance of continuous monitoring of *Vibrio* spp. on plastics to assess their long-term ecological impacts and potential public health risks.

## Data Availability

The raw sequencing data (Fastq files) of 16S rDNA sequencing are available on the Sequence Read Archive public database at NCBI under the project number: PRJNA1218930.

## Supplementary Materials

The following supporting information can be downloaded at: https://www.mdpi.com/article/10.3390/toxics14050392/s1, Figure S1: Monthly variation in the composition of the total bacterial community between expanded polystyrene (EPS) and in seawater; Figure S2: Monthly variation in the composition of the potentially pathogenic bacterial community on expanded polystyrene (EPS) and in seawater. The heatmap illustrates the relative abundance of potentially pathogenic *Vibrio* species; Figure S3: Concentrations of dissolved inorganic nitrogen, dissolved inorganic phosphorus, and chlorophyll-a across sampling periods. Sampling periods are grouped based on Nussbaum clustering using Euclidean distance; Figure S4: Heatmap illustrating the relative abundance of dominant potentially pathogenic bacterial (PPB) community species under combined environmental conditions defined by temperature, dissolved organic carbon (DOC), and eutrophication index (EI), clustered according to categories shown in Figure 3; Table S1: PCR Amplification of the V3–V4 Regions in 16S rDNA; Table S2: Summary of Quality Control for the Divisive Amplicon Denoising Algorithm 2 (DADA2), Including Quality Filtering, Trimming, Denoising, Merging of Paired Reads, and Removal of Chimeric Sequences, as Well as GC Content (%), Q20 (%), Q30 (%), and Coverage Obtained From 16S rDNA Metabarcoding Results. Environmental Factors Are Also Included. (Expanded Polystyrene: EPS); Table S3: Summary of Potential Pathogenic Bacteria and Their Hosts; Table S4: Summary of Discriminative Microbial Features Identified by LEfSe Analysis, Including Bacterial Communities, Taxonomic Groups, Substrate Type, LDA Scores (Log10), and Associated Statistical Significance (*p* value and q value). False Discovery Rate (FDR)-Adjusted q values Are Shown in the Last Column (q value). (Expanded Polystyrene: EPS); Table S5: Statistics of All Taxa Analyzed Using Microbiome Multivariable Association With Linear Models 2 (MaAsLin2). False Discovery Rate (FDR)-Adjusted *p* values Are Shown in the Last Column (q value). (Eutrophication Index: EI, Dissolved Oxygen: DO, Dissolved Organic Carbon: DOC).

## Abbreviations

The following abbreviations are used in this manuscript:

ASV	Amplicon sequence variants
Chl-a	Chlorophyll-a
DADA2	Deficiency of Adenosine Deaminase 2
DIN	Dissolved inorganic nitrogen
DIP	Dissolved inorganic phosphorus
DO	Dissolved oxygen
DOC	Dissolved organic carbon
DOM	Dissolved organic matter
DSi	Dissolved silica
EI	Eutrophication index
EID2 EPS	Enhanced infectious disease database 2 Expanded polystyrene
FDR	False discovery rate
gDNA	Genomic DNA
GOF	Goodness-of-fit
JBTMS	Jangmok Bay Time-series Monitoring Site
KIOST	Korea Institute of Ocean Science & Technology
LEfSe	Linear discriminant analysis effect size
MaAsLin2	Microbiome Multivariable Association with Linear Models 2
mtry	Optimal number of variables per split
NMDS	Non-metric multidimensional scaling
nodesize	Minimum node size
NSEC	Nash–Sutcliffe efficiency coefficient
ntree	Number of trees
R^2^	Coefficient of determination
PCR	Polymerase chain reaction
PERMANOVA	Permutational analysis of variance
PLS-PM	Partial least squares path modeling
PPB community	Potentially Pathogenic Bacterial community
QIIME2	Quantitative Insights Into Microbial Ecology 2
RMSE	Root-mean-square error
SEM	Scanning electron microscopy
TSS	Total sum scaling
VEuPathDB	Eukaryotic Pathogen, Vector & Host Informatics Resources

## References

[1] Statista Research Department Annual Production of Plastics Worldwide from 1950 to 2023 as of June 27, 2025. https://www.statista.com/statistics/282732/global-production-of-plastics-since-1950.

[2] Jambeck J.R., Geyer R., Wilcox C., Siegler T.R., Perryman M., Andrady A., Narayan R., Law K.L. (2015). Plastic waste inputs from land into the ocean. Science.

[3] Xu X., Wang S., Gao F., Li J., Zheng L., Sun C., He C., Wang Z., Qu L. (2019). Marine microplastic-associated bacterial community succession in response to geography, exposure time, and plastic type in China’s coastal seawaters. Mar. Pollut. Bull..

[4] Zettler E.R., Mincer T.J., Amaral-Zettler L.A. (2013). Life in the “plastisphere”: Microbial communities on plastic marine debris. Environ. Sci. Technol..

[5] Sooriyakumar P., Bolan N., Kumar M., Singh L., Yu Y., Li Y., Weralupitiya C., Vithanage M., Ramanayaka S., Sarkar B. (2022). Biofilm formation and its implications on the properties and fate of microplastics in aquatic environments: A review. J. Hazard. Mater. Adv..

[6] Bryant J.A., Clemente T.M., Viviani D.A., Fong A.A., Thomas K.A., Kemp P., Karl D.M., White A.E., DeLong E.F. (2016). Diversity and activity of communities inhabiting plastic debris in the North Pacific Gyre. mSystems.

[7] Zhang S.-J., Zeng Y.-H., Zhu J.-M., Cai Z.-H., Zhou J. (2022). The structure and assembly mechanisms of plastisphere microbial community in natural marine environment. J. Hazard. Mater..

[8] Dang H., Li T., Chen M., Huang G. (2008). Cross-ocean distribution of *Rhodobacterales* bacteria as primary surface colonizers in temperate coastal marine waters. Appl. Environ. Microbiol..

[9] Jeong G., Kim H.-J., Kim K.E., Kim Y.J., Lee T.-K., Shim W.J., Jung S.W. (2023). Selective attachment of prokaryotes and emergence of potentially pathogenic prokaryotes on four plastic surfaces: Adhesion study in a natural marine environment. Mar. Pollut. Bull..

[10] Datta M.S., Sliwerska E., Gore J., Polz M.F., Cordero O.X. (2016). Microbial interactions lead to rapid micro-scale successions on model marine particles. Nat. Commun..

[11] Junaid M., Siddiqui J.A., Sadaf M., Liu S., Wang J. (2022). Enrichment and dissemination of bacterial pathogens by microplastics in the aquatic environment. Sci. Total Environ..

[12] Kim H.-J., Park J.S., Kim S.M., Kim K.E., Kim Y.J., Kim M.-J., Cha H.-G., Hyun B., Shim W.J., Lee S.H. (2025). Effects of environmental factors on the population dynamics of potential pathogenic bacterial community attached to marine plastic debris. J. Hazard. Mater..

[13] Gall S.C., Thompson R.C. (2015). The impact of debris on marine life. Mar. Pollut. Bull..

[14] Galloway T.S., Lewis C.N. (2016). Marine microplastics spell big problems for future generations. Proc. Natl. Acad. Sci. USA.

[15] Carbery M., O’Connor W., Palanisami T. (2018). Trophic transfer of microplastics and mixed contaminants in the marine food web and implications for human health. Environ. Int..

[16] Kim H.-J., Park J.S., Lee T.-K., Kang D., Kang J.-H., Shin K., Jung S.W. (2021). Dynamics of marine bacterial biofouling communities after initial *Alteromonas genovensis* biofilm attachment to anti-fouling paint substrates. Mar. Pollut. Bull..

[17] Kim H.-J., Kim Y.J., Kang D., Kim H., Cho S., Lee T.-K., Lee S.H., Jung S.W., Kang J. (2024). Co-occurrence between key HAB species and particle-attached bacteria and substrate specificity of attached bacteria in the coastal ecosystem. Harmful Algae.

[18] Jung S.W., Joo H.M., Park J.S., Lee J.H. (2010). Development of a rapid and effective method for preparing delicate dinoflagellates for scanning electron microscopy. J. Appl. Phycol..

[19] Bolyen E., Rideout J.R., Dillon M.R., Bokulich A.N., Abnet C.C., Al-Ghalith G.A., Alexander H., Alm E.J., Arumugam M., Asnicar F. (2019). Reproducible, interactive, scalable and extensible microbiome data science using QIIME 2. Nat. Biotechnol..

[20] Callahan B.J., McMurdie P.J., Rosen M.J., Han A.W., Johnson A.J.A., Holmes S.P. (2016). DADA2: High-resolution sample inference from Illumina amplicon data. Nat. Methods.

[21] Álvarez-Jarreta J., Amos B., Aurrecoechea C., Bah S., Barba M., Barreto A., Basenko E.Y., Belnap R., Blevins A., Böhme U. (2024). VEuPathDB: The eukaryotic pathogen, vector and host bioinformatics resource center in 2023. Nucleic Acids Res..

[22] Peterson J.W., Baron S. (1996). Bacterial Pathogenesis. Medical Microbiology.

[23] Austin B., Belkin S., Colwell R.R. (2005). Bacterial pathogens of marine fish. Oceans and Health: Pathogens in the Marine Environment.

[24] European Environment Agency (1999). State and Pressures of the Marine and Coastal Mediterranean Environment.

[25] Primpas I., Tsirtsis G., Karydis M., Kokkoris G.D. (2010). Principal component analysis: Development of a multivariate index for assessing eutrophication according to the European water framework directive. Ecol. Indic..

[26] Anderson M.J. (2001). A new method for non-parametric multivariate analysis of variance. Austral Ecol..

[27] Oksanen J., Simpson G., Blanchet F., Kindt R., Legendre P., Minchin P., O’Hara R., Solymos P., Stevens M., Szoecs E. (2025). Vegan: Community Ecology Package, Version2.7-1.

[28] Wickham H. (2016). Ggplot2: Elegant Graphics for Data Analysis.

[29] Segata N., Izard J., Waldron L., Gevers D., Miropolsky L., Garrett W.S., Huttenhower C. (2011). Metagenomic biomarker discovery and explanation. Genome Biol..

[30] Kruskal W.H., Wallis W.A. (1952). Use of ranks in one-criterion variance analysis. J. Am. Stat. Assoc..

[31] Cao Y., Dong Q., Wang D., Zhang P., Liu Y., Niu C. (2022). microbiomeMarker: An R/Bioconductor package for microbiome marker identification and visualization. Bioinformatics.

[32] Foster Z.S.L., Sharpton T.J., Grünwald N.J. (2017). Metacoder: An R package for visualization and manipulation of community taxonomic diversity data. PLoS Comput. Biol..

[33] Breiman L. (2001). Random forests. Mach. Learn..

[34] Nussbaum M., Spiess K., Baltensweiler A., Grob U., Keller A., Greiner L., Schaepman M.E., Papritz A. (2018). Evaluation of digital soil mapping approaches with large sets of environmental covariates. SOIL.

[35] Mallick H., Rahnavard A., McIver L.J., Ma S., Zhang Y., Nguyen L.H., Tickle T.L., Weingart G., Ren B., Schwager E.H. (2021). Multivariable association discovery in population-scale meta-omics studies. PLoS Comput. Biol..

[36] Tenenhaus M., Vinzi V.E., Chatelin Y.-M., Lauro C. (2005). PLS path modeling. Comput. Stat. Data Anal..

[37] Grömping U. (2006). Relative importance for linear regression in R: The package relaimpo. J. Stat. Softw..

[38] Wang J., Pan F., Soininen J., Heino J., Shen J. (2016). Nutrient enrichment modifies temperature–biodiversity relationships in large-scale field experiments. Nat. Commun..

[39] Wiggin K.J., Chung R.K., Gilbert J.A., Allard S.M. (2025). Effects of temperature and nutrient load on the interaction of *Vibrio parahaemolyticus* and plastic pollution in the marine environment. Mar. Pollut. Bull..

[40] Lo L.S.H., Tong R.M.K., Chan W., Ho W., Cheng J. (2025). Bacterial pathogen assemblages on microplastic biofilms in coastal waters. Mar. Pollut. Bull..

[41] Frère L., Maignien L., Chalopin M., Huvet A., Rinnert E., Morrison H., Kerninon S., Cassone A.-L., Lambert C., Reveillaud J. (2018). Microplastic bacterial communities in the Bay of Brest: Influence of polymer type and size. Environ. Pollut..

[42] Steinberg P.D., de Nys R., Kjelleberg S. (2002). Chemical cues for surface colonization. J. Chem. Ecol..

[43] Yang B., Zhai S., Li X., Tian J., Li Q., Shan H., Liu S. (2021). Identification of *Vibrio alginolyticus* as a causative pathogen associated with mass summer mortality of the Pacific Oyster (*Crassostrea gigas*) in China. Aquaculture.

[44] Kimes N.E., Grim C.J., Johnson W.R., Hasan N.A., Tall B.D., Kothary M.H., Kiss H., Munk A.C., Tapia R., Green L. (2011). Temperature regulation of virulence factors in the pathogen *Vibrio coralliilyticus*. ISME J..

[45] Rodrigues S., Paillard C., Van Dillen S., Tahrioui A., Berjeaud J.-M., Dufour A., Bazire A. (2018). Relation between biofilm and virulence in *Vibrio tapetis*: A Transcriptomic Study. Pathogens.

[46] Song J., Liu X., Wu C., Zhang Y., Fan K., Zhang X., Wei Y. (2021). Isolation, identification and pathogenesis study of *Vibrio diabolicus*. Aquaculture.

[47] Zhang X., Zhang Y., Wu N., Li W., Song X., Ma Y., Niu Z. (2021). Colonization characteristics of bacterial communities on plastic debris: The localization of immigrant bacterial communities. Water Res..

[48] Farto R., Pérez M.J., Fernández-Briera A., Nieto T.P. (2002). Purification and partial characterisation of a Fish lethal extracellular protease from *Vibrio pelagius*. Vet. Microbiol..

[49] Zhang Q., Dong X., Chen B., Zhang Y., Zu Y., Li W. (2016). Zebrafish as a useful model for zoonotic *Vibrio parahaemolyticus* pathogenicity in fish and human. Dev. Comp. Immunol..

[50] Ghosh A., Bhadury P. (2019). *Vibrio chemaguriensis* sp. nov., from Sundarbans, Bay of Bengal. Curr. Microbiol..

[51] Sheikh H.I., Alhamadin N.I.I., Liew H.J., Fadhlina A., Wahid M.E.A., Musa N., Jalal K.C.A. (2024). Virulence factors of the zoonotic pathogen *Vibrio alginolyticus*: A review and bibliometric analysis. Appl. Biochem. Microbiol..

[52] Billaud M., Seneca F., Tambutté E., Czerucka D. (2022). An increase of Seawater temperature upregulates the expression of *Vibrio parahaemolyticus* virulence factors implicated in adhesion and biofilm formation. Front. Microbiol..

[53] Dupke S., Buchholz U., Fastner J., Förster C., Frank C., Lewin A., Rickerts V., Selinka H.-C. (2023). Impact of climate change on waterborne infections and intoxications. J. Health Monit..

[54] Awad D.A., Masoud H.A., Hamad A. (2024). Climate changes and food-borne pathogens: The impact on human health and mitigation strategy. Clim. Change.

[55] Kristjánsson M.M., Magnússon Ó.T., Gudmundsson H.M., Alfredsson G.Á., Matsuzawa H. (1999). Properties of a subtilisin-like proteinase from a psychrotrophic *Vibrio* species. Eur. J. Biochem..

[56] Vezzulli L., Grande C., Reid P.C., Hélaouët P., Edwards M., Höfle M.G., Brettar I., Colwell R.R., Pruzzo C. (2016). Climate influence on *Vibrio* and associated human diseases during the past half-century in the coastal North Atlantic. Proc. Natl. Acad. Sci. USA.

[57] Mizan M.F.R., Jahid I.K., Kim M., Lee K.-H., Lim T.J., Ha S.-D. (2016). Variability in biofilm formation correlates with hydrophobicity and quorum sensing among *Vibrio parahaemolyticus* isolates from food contact surfaces and the distribution of the genes involved in biofilm formation. Biofouling.

[58] Arnórsdóttir J., Smáradóttir R.B., Magnússon Ó.T., Thorbjarnardóttir S.H., Eggertsson G., Kristjánsson M.M. (2002). Characterization of a cloned subtilisin-like serine proteinase from a psychrotrophic *Vibrio* species. Eur. J. Biochem..

[59] O’Malley M.A. (2008). ‘Everything is everywhere: But the environment selects’: Ubiquitous distribution and ecological determinism in microbial biogeography. Stud. Hist. Philos. Biol. Biomed. Sci..

[60] Leighton R.E., Correa Vélez K.E., Xiong L., Creech A.G., Amirichetty K.P., Anderson G.K., Cai G., Norman R.S., Decho A.W. (2023). *Vibrio parahaemolyticus* and *Vibrio vulnificus* in vitro colonization on plastics influenced by temperature and strain variability. Front. Microbiol..

[61] Kesy K., Labrenz M., Scales B.S., Kreikemeyer B., Oberbeckmann S. (2021). *Vibrio* colonization Is highly dynamic in early microplastic-associated biofilms as well as on field-collected microplastics. Microorganisms.

[62] Worden A.Z., Seidel M., Smriga S., Wick A., Malfatti F., Bartlett D., Azam F. (2006). Trophic regulation of *Vibrio cholerae* in coastal marine waters. Environ. Microbiol..

[63] Hoffmann M., Monday S.R., Allard M.W., Strain E.A., Whittaker P., Naum M., McCarthy P.J., Lopez J.V., Fischer M., Brown E.W. (2012). *Vibrio caribbeanicus* sp. nov., isolated from the marine sponge *Scleritoderma cyanea*. Int. J. Syst. Evol. Microbiol..

[64] Liang J., Liu J., Wang X., Lin H., Liu J., Zhou S., Sun H., Zhang X.-H. (2019). Spatiotemporal dynamics of freel-Living and particle-associated *Vibrio* communities in the northern Chinese marginal seas. Appl. Environ. Microbiol..

[65] Zhang X., Zhang Q., Yan T., Jiang Z., Zhang X., Zuo Y.Y. (2015). Quantitatively predicting bacterial adhesion Using surface free energy determined with a spectrophotometric method. Environ. Sci. Technol..

