# Supplementary material for: Dynamics of Attached Bacteria and Potentially Pathogenic Bacteria to Expanded Polystyrene Plastic Litter in Marine Field Experiments
Source: Toxics. 2026 May 2;14(5):392. doi: 10.3390/toxics14050392 (PMC13210944; doi:10.3390/toxics14050392)
Supplement: Supplementary file 1 [file toxics-14-00392-s001.zip › toxics-4245844-supplementary.pdf]

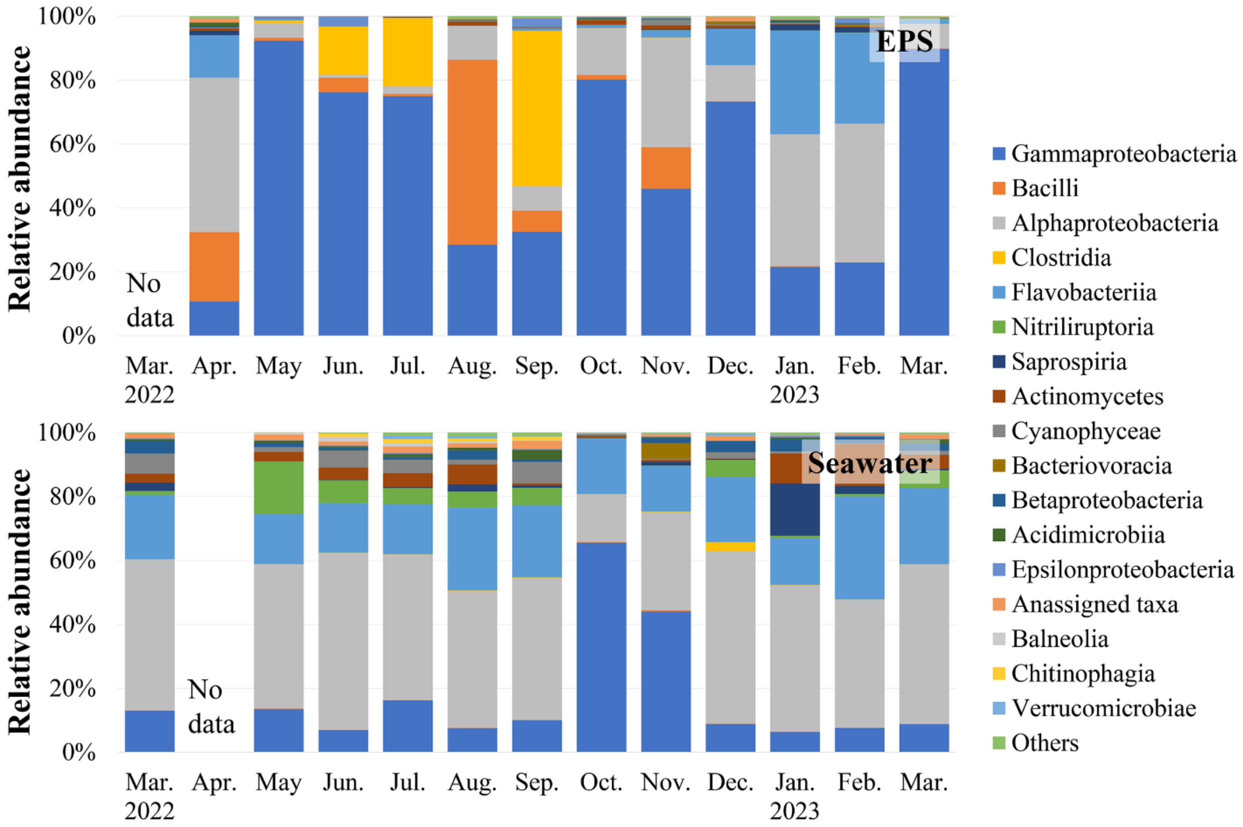

Figure. S1. Monthly variation in the composition of the total bacterial community between expanded polystyrene (EPS) and in seawater.

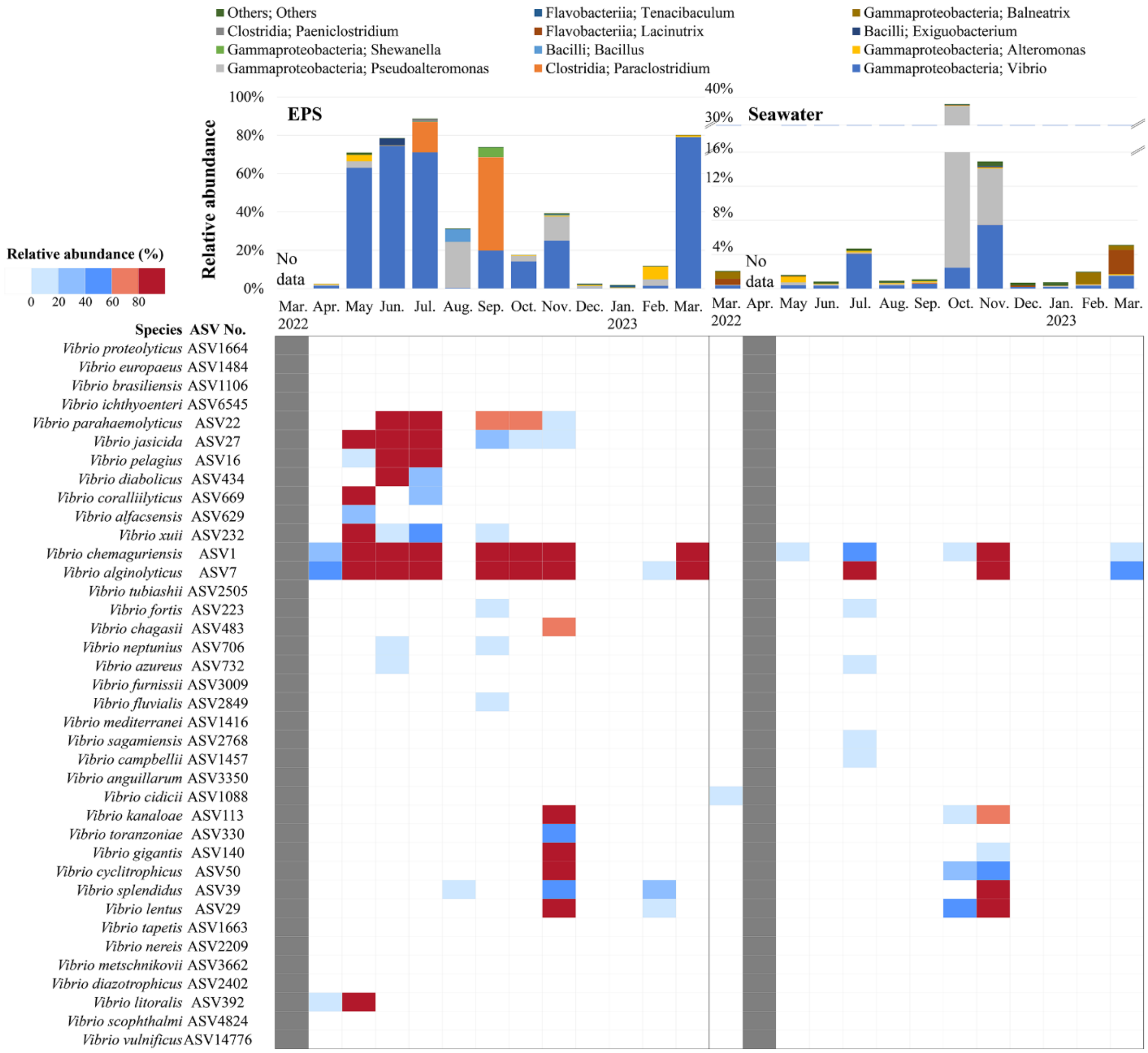

Figure. S2. Monthly variation in the composition of the potentially pathogenic bacterial community on expanded polystyrene (EPS) and in seawater. The heatmap illustrates the relative abundance of potentially pathogenic *Vibrio* species.

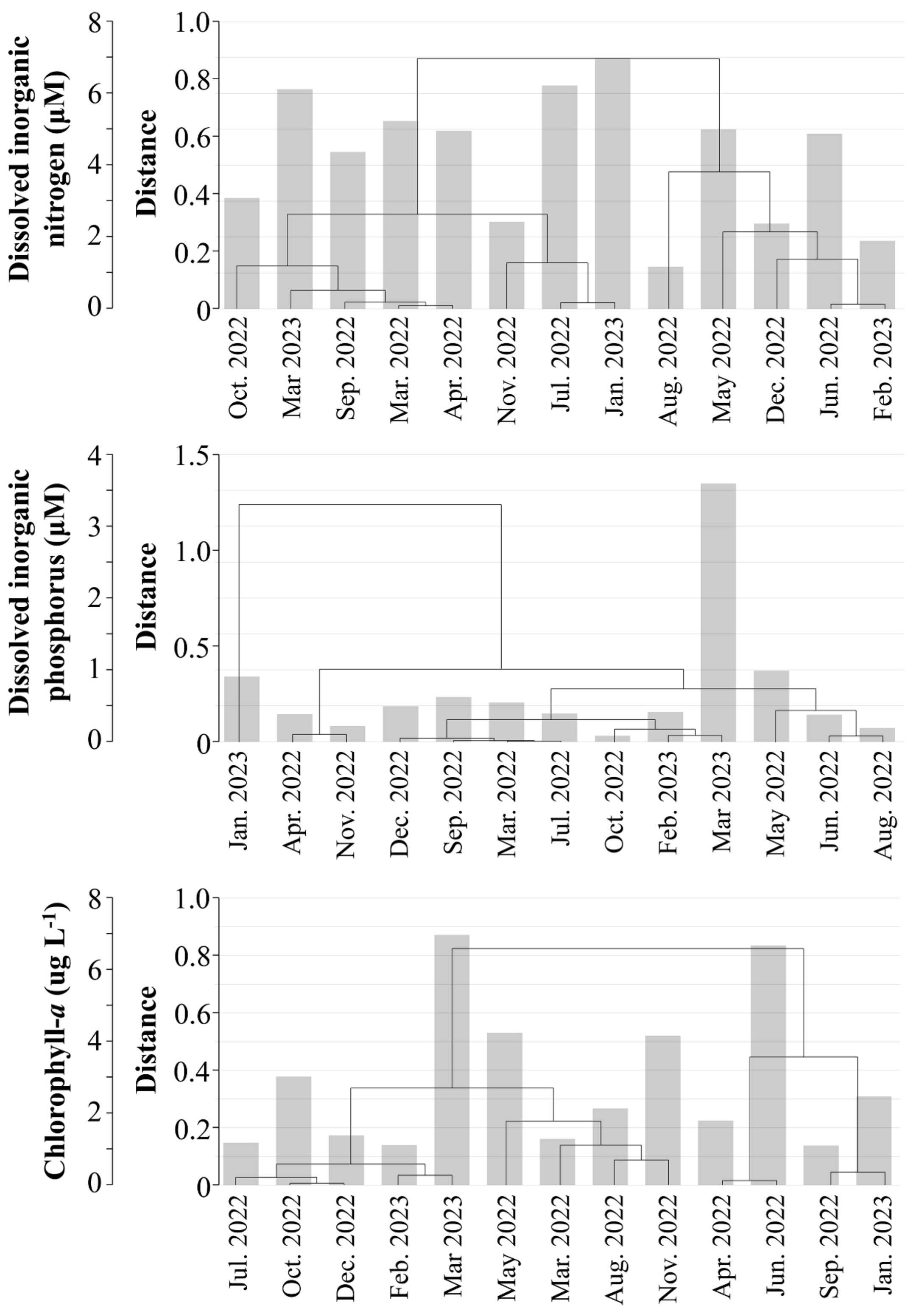

Figure. S3. Concentrations of dissolved inorganic nitrogen, dissolved inorganic phosphorus, and chlorophyll-a across sampling periods. Sampling periods are grouped based on hierarchical clustering using Euclidean distance.

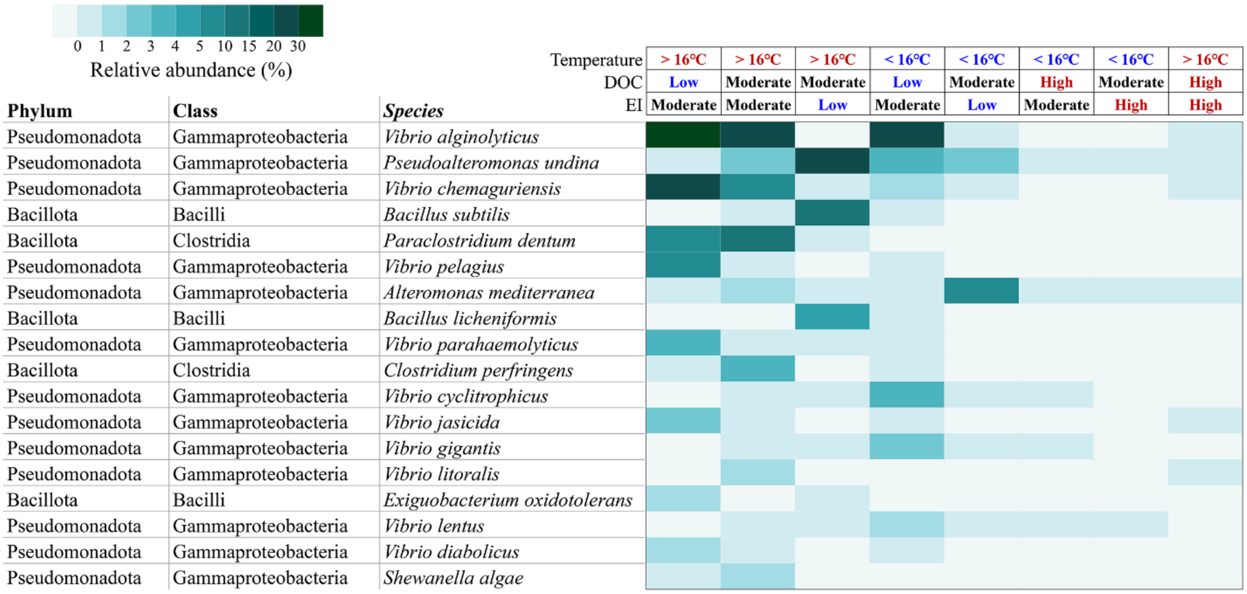

Figure S4. Heatmap illustrating the relative abundance of dominant potentially pathogenic bacterial community (PPB) species under combined environmental conditions defined by temperature, dissolved organic carbon (DOC), and eutrophication index (EI), clustered according to categories shown in Fig. 3.
